# Plant-Forward Dietary Approaches to Reduce the Risk of Cardiometabolic Disease Among Hispanic/Latinx Adults Living in the United States: A Narrative Review

**DOI:** 10.3390/nu18020220

**Published:** 2026-01-10

**Authors:** Franze De La Calle, Joanna Bagienska, Jeannette M. Beasley

**Affiliations:** 1 Department of Nutrition and Food Studies, New York University, New York, NY 10012, USA; 2 Department of Population Health, NYU Grossman School of Medicine, New York, NY 10016, USA; 3 Department of Medicine, NYU Grossman School of Medicine, New York, NY 10016, USA

**Keywords:** Hispanic/Latinx, dietary patterns, plant-forward diet, cardiometabolic risk

## Abstract

**Background**: Cardiometabolic risk (CMR), including obesity, dyslipidemia, hypertension, and impaired glucose regulation, disproportionately affects Hispanic/Latinx adults in the United States (U.S.). Although plant-forward dietary patterns are established as cardioprotective, less is known about how dietary patterns within Hispanic/Latinx subgroups relate to CMR. **Methods**: A narrative review was conducted of observational studies among U.S. Hispanic/Latinx adults (≥18 years) examining defined dietary patterns (a priori, a posteriori, or hybrid) in relation to CMR outcomes (e.g., BMI, waist circumference, blood pressure, glucose, lipids). Risk of bias was assessed using an adapted version of the Newcastle–Ottawa Scale. **Results**: Ten studies met the inclusion criteria, including Seventh-day Adventist Latinx, Puerto Rican adults, Mexican American adults, Hispanic women, and a national Hispanic cohort. Plant-forward dietary patterns were associated with lower BMI and waist circumference, lower triglycerides and fasting glucose, and higher HDL-C. In contrast, energy-dense patterns characterized by refined grains, added sugars, processed meats, fried foods, solid fats, and sugar-sweetened beverages were associated with greater adiposity, poorer lipid profiles, and higher blood pressure. Traditional rice-and-beans–based patterns observed in Puerto Rican and Mexican American groups were associated with central adiposity and higher metabolic syndrome prevalence, despite modestly higher intakes of fruits, vegetables, and fiber. Study quality ranged from good (*n* = 4) to very good (*n* = 6). **Conclusions**: Across Hispanic/Latinx subgroups, plant-forward dietary patterns were associated with favorable cardiometabolic profiles, whereas refined and animal-based patterns aligned with higher CMR. Given the predominance of cross-sectional evidence, these findings should be interpreted as associative rather than causal. Culturally grounded dietary counseling, along with additional longitudinal and intervention studies, is needed to support cardiometabolic health in these populations.

## 1. Introduction

Cardiometabolic risk (CMR) encompasses modifiable factors, including obesity, dyslipidemia, hypertension, and impaired glucose regulation, that increase the likelihood of developing cardiovascular diseases (CVD) [1]. According to the World Health Organization, cardiometabolic diseases (CMD), including CVD and type 2 diabetes mellitus (T2DM), accounted for an estimated 74% of deaths worldwide in 2019 [2]. In the United States (U.S.), approximately one-quarter of adults are affected by CMD, with rates of T2DM and CVD at 11% and 10%, respectively [3]. Additionally, cardiometabolic-related mortality trends in U.S. adults increased from 9.4% to 14.4% from 1999 to 2018, with the largest declines in cardiometabolic health observed for adiposity and glucose-related indicators [4]. These findings underscore the importance of upstream, population-level strategies, including lifestyle, to reduce inequities in cardiometabolic risk.

Evidence shows that individuals with CMD with unhealthy lifestyle practices have a 63% higher risk of mortality than those who maintain a lifestyle concordant with evidence-based guidelines [5]. Diet modification remains a viable strategy for reducing CMR factors and the burden of CMD. There is abundant evidence from randomized trials and observational studies linking key components of a cardioprotective diet, such as fruits, vegetables, whole grains, fish and shellfish, nuts, and vegetable oils, to improvements in cardiometabolic health [6]. Conversely, food components such as hydrogenated oils, processed meats, sugar-sweetened beverages, and refined grains have been well established as having detrimental effects on cardiometabolic health [6].

More recently, there has been a shift from emphasizing individual nutrients, foods, or food groups to identifying combinations of foods, known as dietary patterns, that may better predict health status and disease risk, as their components act synergistically [7]. Over the past two decades, different dietary patterns have been evaluated for their potential beneficial effects on key CMR factors such as body mass index (BMI), blood pressure (BP), glucose regulation, lipid profiles, inflammation, and cardiovascular events [6,7,8]. A broad range of dietary patterns has been well studied, including the Mediterranean, Nordic, traditional Asian, plant-based or vegetarian, and the Dietary Approaches to Stop Hypertension (DASH) [6,8]. In general, there is strong evidence indicating that dietary patterns characterized by higher intakes of minimally processed plant foods (fruits and vegetables), whole grains, legumes, fish, healthy oils, and lower intakes of red and processed meats, refined grains, and added sugars are associated with reduced CMD incidence, progression, and mortality [8]. Moderate evidence further suggests that such patterns reduce the risk for T2DM and support favorable body weight outcomes, including lower BMI, waist circumference (WC), percent body fat, and obesity risk [7]. Conversely, patterns high in red meat, especially from ultra-processed forms [9], and low in fruits and vegetables are linked to poorer cardiometabolic outcomes [10]. Collectively, these findings suggest that the cardiometabolic benefits of dietary patterns depend largely on their overall food composition and emphasis on predominantly minimally processed, plant-forward foods.

Mechanisms underlying the therapeutic effects of healthy dietary patterns on obesity, T2DM, and CVD include reduced energy density and saturated fat content, higher dietary fiber, and the anti-inflammatory properties of plant bioactive compounds [11,12]. Healthful dietary patterns also act through several interconnected biological pathways: regulating nutrient-sensing mechanisms to support glucose and lipid balance, modulating immune homeostasis to reduce inflammation, improving gut microbiome composition, and stabilizing circadian rhythms, all of which contribute to a healthier metabolic profile [8]. Furthermore, minimally processed plant-forward foods supply vitamins, minerals, and antioxidants that support cardiometabolic protection.

Different methodological approaches are used to study dietary patterns, each with distinct strengths and limitations [7,13]. A priori methods, such as index-based approaches, assess adherence to predefined dietary patterns (e.g., the Healthy Eating Index) and offer comparability and ease of use, though they may oversimplify diets and lack specificity. A posteriori, or data-driven methods, including factor, cluster, and principal component analyses, derive patterns empirically from intake data, provide greater detail but often produce population-specific patterns that are difficult to replicate across studies. Hybrid approaches, such as reduced rank regression (RRR), integrate both strategies by deriving patterns based on predictors of specific health outcomes. Despite these methodological differences, dietary pattern analysis captures how combinations of foods and nutrients operate synergistically to influence health outcomes.

To the best of our knowledge, no review has systematically synthesized evidence on dietary patterns among Hispanic/Latinx adults living in the U.S. in relation to cardiometabolic health, despite well-documented disparities in both diet quality and CMR in this population. The purpose of this narrative review is to examine how defined dietary patterns among Hispanic/Latinx adults living in the U.S. relate to key CMR factors. This review compares different dietary patterns characterized within observational studies and highlights their potential to reduce CMR. Guided by this objective, we address the central question: How do defined dietary patterns relate to CMR factors among Hispanic/Latinx adults living in the U.S.?

## 2. Materials and Methods

### 2.1. Search Strategy

A comprehensive systematic search was conducted in partnership with a librarian to ensure that relevant studies were retrieved. The literature search was performed using MEDLINE (1974–present), EMBASE (1974–present), CINAHL, Cochrane Library, Web of Science, Google Scholar, and the Academy of Nutrition and Dietetics Evidence Analysis Library, through August 2025. The PubMed search results can be found in the Supplementary Materials (Table S1). No restrictions were placed on language, publication date, or publication status. The search strategy used a string combination of the following keywords: Hispanic, Latinx, Latino, dietary patterns, diet patterns, diet index, cardiometabolic risk, cardiometabolic disease. Manual searches of reference lists were also conducted to identify relevant studies. The search was updated every six months until publication. Grey literature, including theses and dissertations, was searched through ProQuest and Google Scholar to ensure completeness and to identify unpublished but relevant work. The references of papers that met the inclusion criteria were also searched to identify additional articles.

### 2.2. Inclusion and Exclusion Criteria

The eligibility criteria for this review were informed by our Population, Intervention, Comparison, and Outcome (PICO) research question [14]: Population (P): Hispanic/Latinx (herein used interchangeably) adults aged 18 years or older living in the U.S. Intervention/Exposure (I): Defined dietary patterns identified using a priori, a posteriori, or hybrid methods. Comparison (C): Differences between dietary pattern groups or scores. Outcome (O): Associations with at least one CMR factor. Guided by this framework, we included observational studies (case–control, cross-sectional, longitudinal, and ecological studies) that examined the association between dietary patterns (exposure) and CMR outcomes. Dietary patterns could range from plant-forward approaches such as Mediterranean, Vegetarian, Dietary Approaches to Stop Hypertension (DASH), or Plant-Based Indices to Western or other unhealthy patterns, provided they were clearly defined.

Studies were excluded if they exclusively examined children, adolescents, pregnant individuals, or populations with established end-stage kidney disease or other conditions that substantially modify usual dietary intake. Only studies conducted within the U.S. were included. In addition, all Hispanic Community Health Study/Study of Latinos (HCHS/SOL) cohort publications were excluded, as the first and third authors are collaborating on a forthcoming paper focused specifically on dietary patterns and health outcomes within this cohort.

### 2.3. Study Outcomes

Eligible studies assessed at least one CMR factor. Outcomes included BMI, WC, percentage body fat, High-density lipoprotein cholesterol (HDL-C), low-density lipoprotein cholesterol (LDL-C), glycated hemoglobin A1c (HbA1c), fasting blood glucose (FBG), or BP.

Optimal cardiometabolic values, adapted from O’Hearn et al. and aligned with the American Heart Association, were used as reference thresholds: BMI < 25 kg/m^2^; WC ≤ 88 cm (women) and ≤102 cm (men); FBG < 100 mg/dL; HbA1c < 5.7% and BP < 120/80 mmHg [4].

### 2.4. Data Collection and Analysis

All articles were downloaded into EndNote 21.4 and then imported into Covidence for screening and duplicate removal [15]. Two independent reviewers (FDLC and JB) screened all titles and abstracts using the inclusion and exclusion criteria to identify potentially eligible articles. When eligibility remained uncertain, both reviewers (FDLC and JB) independently completed a full paper review and resolved discrepancies through discussion.

### 2.5. Data Extraction and Synthesis

Data were extracted on study characteristics (first author, publication year, study design, data source, data collection years), population details (sample size, age range, sex distribution, Hispanic/Latinx subgroup when specified), and dietary pattern assessment (type of pattern, methodological approach, dietary data collection method, and reference standards used). Dietary pattern labels were reported as originally described by authors (e.g., “Vegetarian”, “Traditional”). Extracted cardiometabolic outcomes included BMI, WC, BP, FBG, HbA1c, lipid biomarkers, and other related indicators of CMR. Key results from each study were documented.

Studies were organized and tabulated by specific Hispanic/Latinx heritage as defined by the original authors, including Mexican American adults, Puerto Rican adults, Hispanic women, Seventh-day Adventist (SDA), and multicultural Hispanics. This allowed for clearer synthesis of dietary patterns within each distinct population and enabled within-group summarization. Adjusted results were extracted whenever available; unadjusted findings were only used when adjusted analyses were not reported. Forest plots were constructed for outcomes with comparable exposure contrasts and sufficient variance reporting. Outcomes reported without measures of uncertainty were summarized narratively or presented as point estimates without pooled effects. In the narrative synthesis, only statistically significant associations were presented unless otherwise noted.

### 2.6. Quality Assessment

Risk of Bias (RoB) was assessed independently by two authors (F.D.L.C and J.B) using an adapted version of the Newcastle–Ottawa Scale for cross-sectional studies (description of the scale found in the Supplementary Materials S2) [16]. This tool evaluates potential RoB across three domains: (1) study sample selection (i.e., participants), (2) comparability of groups, and (3) outcome assessment. Each item is rated using a star system that reflects whether appropriate methodological approaches were used, allowing us to determine the likelihood of bias within each study. The overall RoB score was calculated by summing the stars awarded across all domains. Studies receiving 9–10 stars were classified as “very good quality”, those with 7–8 stars as “good quality”, 5–6 stars as “satisfactory quality”, and 0–4 stars as “unsatisfactory quality”.

## 3. Results

**Figure 1:**
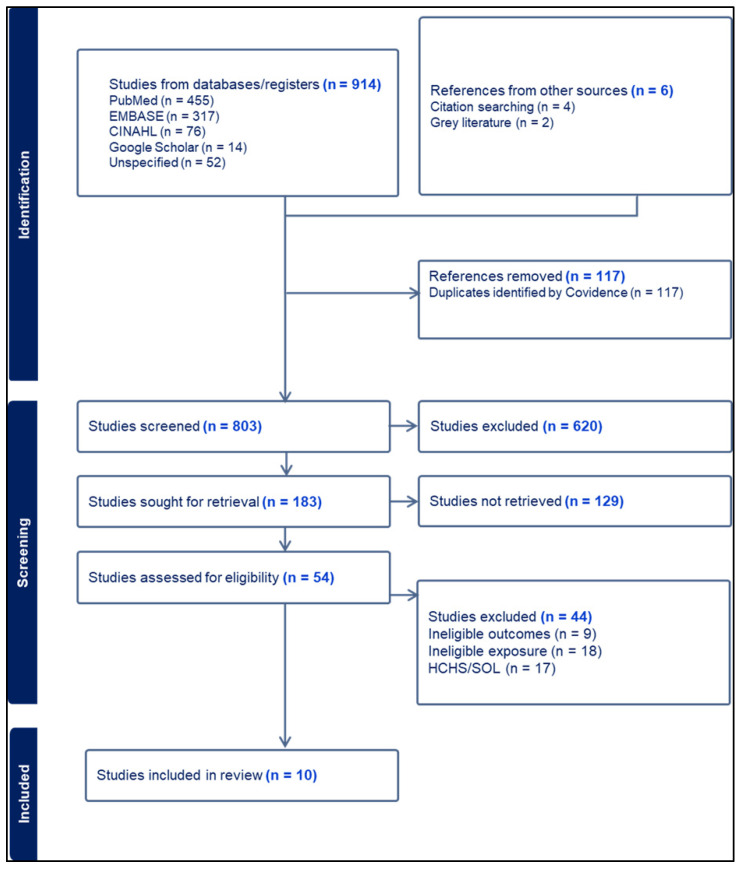
PRISMA flowchart.

The study selection process is presented in Figure 1. A total of 914 records were identified through database searches, including PubMed, EMBASE, CINAHL, Google Scholar, and other sources, with an additional 6 records retrieved through citation searching and gray literature. After removing 117 duplicate records, 803 studies remained for title and abstract screening. Of these, 620 records were excluded for not meeting the inclusion criteria. Full texts of 183 articles were sought; 129 were excluded, leaving 54 assessed for eligibility. Of these, 44 were excluded (list of titles can be found in the Supplementary Materials S3). Ultimately, 10 studies met all inclusion criteria and were included in the final extraction process.

### 3.1. Risk of Bias

Overall, most studies demonstrated strong quality based on the Newcastle–Ottawa Scale (Table 1). Six studies were rated “very good” (scores 9–10), reflecting well-defined sample selection, adjustment for key confounders, use of validated dietary assessment tools, and objective clinical outcomes that were not self-reported [17,18,19,20,21,22]. The remaining studies were rated “Good” (scores 8), generally due to self-reported outcomes or lack of non-response comparisons [23,24,25,26]. Systematic sampling was common in the National Health and Nutrition Examination Survey (NHANES), the Boston Puerto Rican Health Study (BPRHS), and cohort-based studies, whereas several church or community-recruited samples used convenience methods. Across studies, dietary instruments were consistently validated (Food frequency questionnaires (FFQs) or multiple 24 h recalls, and in some instances, outcome measures were clinically verified using blood biomarkers.

### 3.2. Sample Characteristics

The sociodemographic and health characteristics of the sample population are presented in Table 2. Across studies, adults adhering to plant-based or vegetarian dietary patterns tended to be older, more often women, more highly educated, and more likely to have healthier lifestyle profiles. For example, in Singh et al., 2019, SDA vegetarians were more often women at 82.6% vs. 66% and more likely to have higher education at 73.9% vs. 45.1% compared with non-vegetarians [22]. Similarly, in Jaceldo Siegl et al., 2019, adults in the plant-based group were more often never smokers at 84.2% vs. 80.8% and more likely to have higher education at 42.6% vs. 33.8% [23]. In the nationally representative NHANES sample analyzed by Osborn and Haemer 2023, individuals in the highest tertile of the plant-based pattern were also more likely to have some college education (51.0%) compared with lower tertiles (43.6%) and with those in the highest tertile of the solid fats and refined carbohydrates pattern (42.1%) [21].

In contrast, energy-dense or animal-based dietary patterns were more common among younger adults, those with lower educational attainment, and those with higher-risk lifestyle behaviors. For example, in Noel et al., 2009, adults in the meat dietary pattern group were younger, with a mean age of 54.6 years compared with 59 years in the traditional PR pattern, had higher rates of smoking (37.2% vs. 23.9%) and alcohol use (53.2% vs. 33.5%) [18]. Similarly, in Arias Gastelum et al., 2021, Hispanic women adhering to high sugar and fat or meat and snack patterns were younger, as indicated by significantly negative associations with age (β = −0.230 and β = −0.298, respectively) [26].

Traditional dietary patterns high in rice and beans were associated with older age, lower education, lower acculturation, and a greater preference for Spanish language use. In Noel et al., 2009, Puerto Rican adults in the traditional PR pattern were the oldest group at 59 years compared with 56.9 years in the sweets pattern and 54.6 years in the meat pattern, and had the highest proportion with less than an eighth-grade education (59.4% vs. 52.3% and 41.6%) [18]. They also showed the lowest acculturation score at 19.2 compared with 24.1 in the sweets pattern and 24.6 in the meat pattern [18]. Similar trends appeared in Reininger et al., 2017, where higher HEI scores, reflecting greater intake of foods such as beans, vegetables, whole grains, and chicken or fish, were observed among Spanish speaking adults (HEI:5.79) compared with bilingual adults (HEI:5.21) and English speakers (HEI: 5.00), and among first generation adults (HEI: 5.67) compared with second generation (HEI: 5.34) and third generation (HEI: 5.59) participants [25].

### 3.3. Study Characteristics

The ten studies included in this review varied in the population assessed and CMR outcomes but together provided a rich view of dietary patterns among Hispanic/Latinx adults in the United States (Table 3) [17,18,19,20,21,22,23,24,25,26]. Three studies were conducted among SDA Hispanic/Latinx adults and primarily compared vegetarian or plant-based dietary (PBD) practices with non-vegetarian patterns [17,22,23]. Three studies focused on Puerto Rican adults, two using the BPRHS cohort aged 45–75 years and one from the Behavioral Risk Factor Surveillance System (BRFSS), examining a community sample in New York City [18,19,24]. Two studies examined Mexican American adults: one using NHANES data (2001–2002) and another using the Cameron County Hispanic Cohort in the Texas-Mexico border, with culturally adapted dietary recall measures from the Physical Activity and Nutrition (SPAN) program to derive Healthy (HEI) and Unhealthy (UEI) Eating Index patterns [20,25]. One study analyzed overweight or obese Hispanic women with diabetes or prediabetes using a Southwestern FFQ to derive six dietary patterns [24]. The remaining study used nationally representative NHANES (2013–2018) data to identify three patterns across a multicultural Hispanic adult population [21].

All studies reported cross-sectional analyses, with one incorporating a prospective follow-up [25]. Across studies, dietary recalls were primarily collected using validated FFQs or 24 h recalls, and dietary patterns were derived through factor analysis [18,21,26], cluster analysis [20], using the European Prospective Investigation into Cancer and Nutrition (EPIC) Oxford cohort dietary patterns [22], predetermined dietary patterns based on intake (e.g., vegetarian, lacto/ovo-vegetarian) [17,23,24], predefined indices [25], or RRR [19].

Sample sizes varied widely, ranging from small community-based cohorts such as in the Singh et al., 2019 and Alexander et al., 1999 articles (*n* = 74 each) [17,22] and Arias-Gastélum et al., 2021 (*n* = 191) [26], to medium-sized Puerto Rican and Mexican American cohorts including Noel et al., 2009 (*n* = 1167) [18], Del Campo et al., 2020 (*n* = 703) [19], Melnik 2006 (*n* = 1304) [24], and Reininger et al., 2017 (*n* = 1250) [25]. The largest datasets were Jaceldo-Siegl et al., 2019 (*n* = 3475) [23] and Osborn and Haemer 2023 (*n* = 2049) [21].

### 3.4. Association Between Dietary Patterns and CMR Outcomes

#### 3.4.1. Adiposity: BMI, and Central Adiposity (WC)

**Figure 2:**
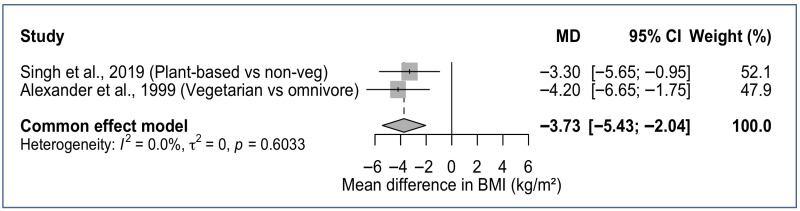
Forest plot of BMI differences comparing plant-based versus omnivorous dietary patterns in SDA Hispanic/Latinx Adults. MD, Mean Difference; CI, Confidence Interval [17,22].

Eight of the ten studies assessed BMI and/or WC [17,19,20,21,22,23,25]. Dietary patterns higher in plant foods consistently aligned with lower BMI and reduced obesity, especially among SDA Latinx (Figure 2). In the AHS-2 cohort (*n* = 3475), BMI was inversely associated with plant-based adherence such that vegans had ~14% lower BMI, lacto-ovo vegetarians ~8% lower, and pesco-vegetarians ~4% lower compared with nonvegetarians [23]. Obesity prevalence ranged from 6.9% in vegans to 28.2% in nonvegetarians, even after adjustment for sociodemographic and behavioral factors [23]. A similar stepwise pattern was observed for overweight BMI (%): vegan (21.3) < vegetarian (31.9) < pesco-vegetarian (36.2) < semi-vegetarian (37.4) < nonvegetarian (38.9) [23]. A smaller cohort of 74 SDAs, comparing a predominantly vegetarian SDA group with omnivore Catholics, showed similar trends; SDAs had a lower mean BMI (27.2 vs. 31.4 kg/m^2^) and smaller WC (89.2 cm vs. 99.3 cm) than omnivorous Catholics [17].

Among Mexican Americans in NHANES 2001–2002, four a posteriori patterns: “poultry and alcohol”, “milk and baked products”, “traditional Mexican”, and “meat”, showed similar BMI values (~28 kg/m^2^), indicating no meaningful BMI differences across patterns [20]. However, WC displayed a stepwise increase among women, with all pattern groups exceeding the 88 cm metabolic-risk threshold: meat (95.2 cm) > poultry and alcohol (94.2 cm) > traditional Mexican (93.1 cm) > milk and baked products (92.0 cm) [20]. Among men, WC followed a different ordering and remained below the ≤102 cm metabolic-risk threshold, with traditional Mexican highest (97.8 cm), followed by poultry and alcohol (95.8 cm), milk and baked products (95.6 cm), and meat (94.2 cm) [20]. In another study of 1250 Mexican American adults from the Cameron County Hispanic Cohort, BMI status was not a significant predictor of consuming foods on the HEI or UEI [25].

Among Puerto Ricans from the BPRHS cohort, both a “meat, processed meat, and French fries” pattern and a “traditional rice, beans, and oils” pattern were associated with higher WC (~102 cm) [18]. Similarly, in a RRR analysis among Puerto Ricans with T2DM, WC increased across tertiles (T1: 99.4 → T2: 104 → T3: 108 cm, *p* = 0.003), reflecting greater central adiposity. In this sample there was higher adherence to a pattern characterized by higher intake of white bread, meat, processed meat, pizza, pasta, Mexican foods, diet soda, sweetened beverages, and certain vegetables (corn, string beans, onions, okra, cucumber, avocado/guacamole), and lower intake of nuts and seeds, reduced-fat dairy, starchy vegetables, soups, and hot cereal [19].

Within NHANES 2013–2018 data (*n* = 2049), a dietary pattern high in solid fats, cheese, and refined carbohydrates was significantly associated with higher BMI at moderate intake (middle vs. lowest tertile β ≈ +1.07 kg/m^2^) and was more commonly consumed by younger, male, Mexican American participants [21]. In contrast, a vegetable pattern rich in red/orange, green, and other vegetables was inversely associated with percent body fat (β ≈ −1.57%) and was more frequently reported among women and those from higher-income households [21].

Among overweight/obese Hispanic women with diabetes/prediabetes, no significant associations emerged between six identified patterns (including a “plant foods and fish” pattern) and BMI or WC, likely reflecting extremely high adiposity at baseline (mean BMI ≈ 36 kg/m^2^; 86% obesity) [26].

Overall, Latinx SDA studies provided the clearest evidence that greater adherence to PB dietary patterns is associated with lower BMI and smaller WC. In contrast, dietary patterns characterized by refined-carbohydrate, high-fat, meat-heavy, or oil-rich foods observed in broader Hispanic adults, including Puerto Rican and Mexican American groups, were consistently linked to higher BMI and larger WC.

#### 3.4.2. Blood Pressure (BP)

BP patterns varied by subgroup and were assessed in half the studies [17,18,19,21,22]. In the SDA and Catholic Hispanics sample, SDA, who had a greater proportion of vegetarians compared to 100% omnivore Catholics, had lower systolic BP (SBP) compared with Catholic omnivores (109.8 vs. 117.1 mmHg), though differences attenuated after adjusting for WC [18]. In this cohort, WC was the strongest independent predictor, accounting for 28% of the variability in SBP and 14% in DBP [17]. In contrast, Singh et al. reported no significant differences in SBP or DBP between vegetarian and nonvegetarian SDAs [22].

In Puerto Rican adults, adherence to the “meat, processed meat, and French fries” pattern predicted higher SBP (136.7 vs. 133.0 mmHg, *p* = 0.002) and DBP (82.2 vs. 78.4 mmHg, *p* = 0.001) across extreme quintiles (Q5 vs. Q1) [18]. Other patterns, such as sweets/desserts, did not strongly influence BP [18]. Del Campo similarly found that among 703 Puerto Rican adults, those with T2DM most adherent to the “diabetes dietary pattern” (characterized by higher intake white bread, processed meats/meats, sweetened beverages, soft drinks; and low in nuts/seeds, reduced-fat dairy, starchy vegetables, reduced-fat dairy) showed a nonsignificant gradual increase in SBP (135 → 138 → 140 mmHg across tertiles). By contrast, the “no-diabetes” pattern was not meaningfully related to SBP, with values remaining fairly stable (130–132 mmHg) [19]. Conversely, in the national multicultural Hispanic NHANES cohort, a high–solid-fat/refined-carbohydrate pattern was unexpectedly associated with lower SBP (β = −2.47 [95% CI: −4.89, −0.59]), despite its unfavorable associations with BMI [21].

#### 3.4.3. Lipid Profile: HDL-C, LDL-C, and Triglycerides

Four of the ten studies examined associations between dietary patterns and lipid biomarkers [17,18,19]. Among Hispanic SDA, triglycerides were considerably lower (152 vs. 229 mg/dL) and HDL-C was higher (44.5 vs. 39.9 mg/dL) compared with Catholic omnivores, indicating a more favorable lipid profile in the SDA group [17]. LDL-C, however, did not differ significantly between groups [17]. Notably, Catholic omnivores reported substantially higher intakes of cholesterol and saturated fat [17], which may partially explain their less favorable lipid measures.

In Puerto Rican adults, higher adherence to the “sweets” pattern, rich in candy, sugary drinks, and desserts, and low in fish, poultry, and vegetables, was significantly associated with lower HDL-C when comparing the highest versus lowest intake levels [18]. Similarly, among a cohort of Puerto Ricans with and without T2DM, those without diabetes who were more adherent to the “non-diabetes dietary pattern” (high in white bread, solid fats, sweet baked goods, processed meat/meat, rice: and low in nuts/seeds, poultry, water) was linked to lower HDL-C (T3: 43.9 vs. T1: 50.8 mg/dL, *p* = 0.01) [19]. Over two years of follow-up, LDL-C declined among individuals with diabetes (−4.44 mg/dL), while HDL-C increased modestly among those without diabetes (+1.03 mg/dL) [19]. However, baseline dietary pattern did not meaningfully predict 2-year changes in HDL-C, LDL-C, or triglycerides among adults with diabetes [19]. Consistent with these findings, Melnik et al. reported that, among New York City Puerto Ricans, a diabetes diagnosis was associated with lower fat intake behaviors (β = −0.27, *p* = 0.01), including modifying meats to be low in fat [24].

In the multicultural Hispanic cohort in NHANES, the solid-fat/refined-carbs pattern predicted lower HDL-C at high tertiles (β = −4.53 [95% CI: −7.03, −2.03]); Vegetable-rich patterns were also linked to lower HDL-C at moderate intake (β = −2.62 [95% CI: −4.79, −0.47]) [21].

Overall, dietary patterns high in sweets, refined grains, and solid fats were consistently associated with lower HDL-C across Hispanic subgroups, whereas vegetarian/SDA diets were associated with more favorable HDL-C and triglyceride profiles.

#### 3.4.4. Glycemic Outcomes, Type 2 Diabetes and Metabolic Syndrome

Plant-forward patterns generally supported healthier glycemic profiles. SDA exhibited much lower FBG compared to Catholic omnivores (88.9 vs. 103.4 mg/dL) [17]. Among Puerto Ricans, those in the highest quintile of the sweets/desserts dietary pattern had a notably lower unadjusted prevalence of diabetes (28.5%) compared with individuals in the highest quintile of the traditional PR (42.5%) and meat (40.9%) dietary patterns. This group also demonstrated significantly lower FBG than those in the lowest quintile (Q5: ~115 mg/dL vs. Q1: ~122 mg/dL); these converted fasting glucose values (mg/dL) are reported in the text only and are not presented in Table 3 [18].

Surprisingly, among overweight or obese Hispanic women with diabetes or prediabetes, adherence to the “plant/fish” pattern was associated with a modest increase in FBG (β ≈ +0.152) [26]. Likewise, among Puerto Ricans, stronger adherence to the “traditional PR” dietary pattern was linked to higher MetS odds (OR ≈ 1.7, 95% CI 1.04–2.7), which increased after excluding individuals with diabetes (OR = 2.5, 95% CI: 1.4–4.4) [18]. Consistent with these findings, those most adherent to the traditional PR pattern had the highest crude MetS prevalence (74.5%), compared with 64.9% in the sweets/desserts pattern and 62.1% in the meat pattern, along with the previously noted prevalence of diabetes burden [18]. Among 1250 Mexican American adults from the Cameron County Hispanic Cohort, diabetes status was not significantly associated with consuming foods on the HEI or UEI [25].

Across subgroups, SDA vegetarians showed the most favorable composite cardiometabolic profile (lower BMI, lower WC, better lipid profiles), indicating a substantially lower overall cardiometabolic burden relative to omnivorous Hispanic counterparts.

## 4. Discussion

This narrative review synthesizes evidence from ten observational studies examining dietary patterns and CMR among diverse Hispanic/Latinx adults in the United States. Despite variation across the studies, methodological approach, and the dietary patterns identified, a consistent theme emerged: dietary patterns rich in plant foods, particularly those observed among SDA, were associated with better cardiometabolic health, including lower BMI and WC, healthier lipid profiles, and more favorable glycemic markers. These findings align with long-standing epidemiologic research and experimental studies demonstrating the protective role of plant-forward, minimally processed diets on cardiometabolic health and reinforce dietary recommendations emphasized for decades in public health and clinical nutrition [27,28]. However, it is important to recognize that the favorable health outcomes observed among SDAs are influenced by more than dietary practices alone. As shown in the studies reviewed, SDA participants also tend to lead generally healthier lifestyles, engaging in regular physical activity and minimizing smoking and alcohol use, which likely contributes to their overall cardiometabolic advantage [29].

In contrast, in this review we found that energy-dense patterns characterized by sweets, refined grains, processed meats, fried foods, solid fats, and sugar-sweetened beverages, commonly observed among Puerto Rican, Mexican American, and nationally representative multicultural Hispanic cohorts, were associated with lower HDL-C, higher triglycerides, higher adiposity, and, in some cases, elevated BP and MetS [18,21]. These associations persisted even after adjustment for sociodemographic and lifestyle factors, suggesting that dietary composition remains a meaningful and independent contributor to cardiometabolic risk. Similar findings were reported in a large prospective cohort of US adults, including a 32-year study of 205,776 U.S. healthcare professionals in which processed meats, red meat, French fries, eggs, tomatoes, poultry, and both low- and high-energy beverages were positively associated with major chronic disease [30].

Interestingly, among Hispanic/Latinx adults, the “traditional” Puerto Rican rice-and-beans dietary pattern was associated with greater central adiposity and a higher prevalence of MetS [18]. Individuals most adherent to this pattern were less acculturated, had lower educational attainment, and were more likely to live below the poverty level. A comparable association was observed among Mexican Americans, where adherence to a “traditional” Mexican dietary pattern was linked to central adiposity, particularly among women [20]. In contrast to the Puerto Rican findings, individuals adhering to the traditional Mexican pattern were more likely to report speaking only English (57%).

Although these traditional patterns were characterized by the highest intakes of fruits, vegetables (traditional Mexican: 3.6–6.4% of total energy), and fiber (traditional Mexican: 23.3 g, and traditional PR: 22 g) within their respective cohorts, absolute intake levels remained below recommended thresholds for a healthful dietary pattern [30]. Furthermore, among Puerto Rican adults, fat-lowering dietary behaviors were more frequently reported by individuals with diabetes but were less common among those with obesity (BMI > 30 kg/m^2^) or a family history of diabetes [24].

Taken together, these findings underscore the complexity of interpreting “traditional” dietary patterns, which appear to reflect not only food choices but also broader social and structural contexts, including socioeconomic disadvantage, acculturation, food access, cultural norms, and family history of chronic disease risk [31,32,33].

Methodologically, all studies were of good to very good quality, used validated dietary assessment tools (FFQs or multiple 24 h recalls), and adjusted for major confounders. However, reliance on cross-sectional designs and self-reported data increases the likelihood of information and sampling bias, including socially desirable answers [34,35]. Heterogeneity in pattern derivation, small SDA cohorts, and convenience sampling also limit generalizability [34]. Nonetheless, the consistency of associations across multiple Hispanic populations and the methodological strategies used across the studies strengthen confidence in the overall findings.

A key strength of this review is its inclusion of diverse Hispanic/Latinx heritage, allowing examination of how dietary patterns relate to multiple CMR factors across Hispanic subgroups. The heterogeneity observed in our study underscores the importance of exploring cultural, generational, and socioeconomic contexts. Puerto Rican, Mexican American men and women, as well as SDA cohorts, revealed varying dietary–risk profiles, which warrant further investigation. However, cross-sectional study designs limit the ability to assess incidence or causality. Longitudinal data was limited in this study, and when examined, baseline dietary patterns did not consistently predict changes in CMR factors over two years, particularly among individuals with diabetes who may receive more intensive medical management over time.

## 5. Conclusions

Taken together, these findings reaffirm long-standing nutritional guidance: Diets emphasizing vegetables, legumes, whole grains, and reduced intake of processed meats, refined carbohydrates, and added sugars support healthier cardiometabolic profiles. Recent meta-analyses and umbrella reviews indicate that healthful plant-based diets are associated with lower adiposity, improved lipid profiles, and reduced cardiometabolic risk, while plant-based diets high in refined carbohydrates confer fewer benefits [36]. This distinction highlights the importance of diet quality within plant-forward eating patterns. For Hispanics/Latinx, the results highlight the value of culturally grounded dietary counseling that honors traditional foodways while adapting them to improve diet quality that aligns with dietary guidelines. Given the predominance of cross-sectional evidence, these findings should be interpreted as associative rather than causal. Future research should prioritize longitudinal and intervention studies among understudied Hispanic/Latinx subgroups to clarify causal pathways, incorporate measures of food insecurity and acculturation, and examine structural factors influencing dietary intake and participation in nutrition interventions. Ultimately, improving cardiometabolic health among Hispanic/Latinx communities will require approaches that respect cultural dietary traditions, address social and environmental barriers, and build upon established evidence-based nutrition practice.

## Figures and Tables

**Table 1:** Newcastle–Ottawa Scale (NOS) quality assessment of included studies (*n* = 10).

First Author, Year	Selection (Max 5)	Comparability (Max 2)	Outcome (Max 3)	Total	Quality	Notes (Sampling, Adjustment, Validation)
Singh et al., 2019 [22]	4	2	3	9	Very Good	Non-random sampling recruited from SDA churches; adjusted models (age, sex, education); validated Epic Oxford FFQ; objective outcomes not self-reported; no non-response comparison.
Alexander et al., 1999 [17]	4	2	3	9	Very Good	Non-random convenience sampling through churches; adjusted models (age, sex, BMI, WC); validated FFQ; objective outcomes not self-reported (anthropometric/labs); no non-respondent data.
Jaceldo-Siegl et al., 2019 [23]	4	2	2	8	Good	Cohort from Adventist Health Study 2 (AHS-2); adjusted models (age, sex, lifestyle); validated FFQ; self-reported outcome (BMI). No non-respondent data.
Noel et al., 2009 [18]	5	2	3	10	Very Good	Sample from BPRHS using block enumeration; fully adjusted models (SES, behaviors, medications); validated FFQ; objective biomarkers; reasons for exclusions/dropouts fully described.
Del Campo, 2020 [19]	5	2	3	10	Very Good	Sample from BPRHS; multivariable adjustment (age, SES, lifestyle); validated FFQ; objective biomarkers; comparison between included/excluded.
Melnik et al., 2006 [24]	4	2	2	8	Good	Cohort from BRFSS using random-digit dialing; adjusted models (age, SES, lifestyle); validated diet behavior tool; outcomes self-reported; no non-response comparison.
Carrera et al., 2007 [20]	4	2	3	9	Very Good	NHANES probability sampling; adjusted models (age, sex, SES); validated 24 h recall; objective anthropometrics; non-response data not reported.
Reininger et al., 2017 [25]	4	2	2	8	Good	Sample from Cameron County Hispanic Cohort (random); adjusted models (age, sex, education); School Physical Activity and Nutrition (SPAN) recall; objective outcomes not self-reported (BMI); no non-respondent data.
Arias-Gastélum et al., 2021 [26]	4	1	3	8	Good	Sample from “De Por Vida” RCT (random); unadjusted analyses (correlations only); validated FFQ; objective biomarkers; no non-respondent data.
Osborn & Haemer, 2023 [21]	4	2	3	9	Very Good	NHANES probability sampling; multivariable adjustment (age, sex, SES, diet, lifestyle); validated 24 h recalls; objective biomarkers; exclusions not compared.

Adapted NOS scores for cross-sectional studies: Very Good: 9–10 points. Good: 7–8 points. Satisfactory: 5–6 points. Unsatisfactory: 0 to 4 points.

**Table 2:** Study population sociodemographic and health characteristics grouped by heritage (*n* = 10).

First Author, Year	Inclusion/Exclusion Criteria	Sample Characteristics
**Seventh-day Adventist Hispanic/Latinx Adults**		
Singh et al., 2019 [22]	Incl. ≥18 y; baptized SDA; Hispanic/Latinx|Excl. Dementia; preg./BF	Veg vs. non-veg: age ~54 vs. 48 y; women 82.6% vs. 66.0%; >college 73.9% vs. 45.1%; foreign-born 87.0% vs. 92.2%; religiosity similar
Alexander et al., 1999 [17]	Incl. Hispanic/Latinx; SDA or Catholic|Excl. Lipid/T2DM meds	Age ~42 (SDA) vs. 45 y (Catholic); religiosity similar; alcohol 0% vs. 42%; smoking 0% vs. 9.5%; PA ~2×/wk
Jaceldo-Siegl et al., 2019 [23]	Incl. SDA Hispanic/Latinx adults (2002–2007)|Excl. Incomplete FFQ/anthro/lifestyle data	Age ~50 y across DPs; women: semi/pesco-veg 72% vs. non-veg 65.6%; >college: PBD 42.6% vs. non-veg 33.8%; foreign-born: PBD 63.8% vs. non-veg 58%|PBD more often never-smokers (84.2% vs. 80.8%) and never-drinkers (66.3% vs. 51.2%) than non-vegetarian
**Puerto Rican Adults**		
Noel et al., 2009 [18]	Incl. PR adults 45–75 y|Excl. Implausible FFQ; serious illness; no permanent address; relocation within 2 years	Highest quantile (Q5) of all DPs: Age (y): Trad PR (59) > Sweets (56.9) > Meat (54.6); Female (%): Sweets (76.2) > Trad PR (75.3) > Meat (53.2); <8th-grade edu (%): Trad PR (59.4) > Sweets (52.3) > Meat (41.6); Acculturation ^†^: Meat (24.6) > Sweets (24.1) > Trad PR (19.2).|Current smoker (%): Meat (37.2) > Sweets (30.0) > Trad PR (23.9); Current alcohol use (%): Meat (53.2) > Sweets (34.6) > Trad PR (33.5).
Del Campo et al., 2020 [19]	Incl. PR adults 45–75 y|Excl. Missing data; antilipemic meds; implausible intake	Highest tertile (T3) of DP scores (T2DM vs. No-T2DM patterns): Age (y): T2DM (55.9) > No-T2DM (52.6); Female (%): No-T2DM (56) < T2DM (60); <8th-grade edu (%): T2DM (45) > No-T2DM (37); BMI (kg/m^2^): T2DM (33.5) > No-T2DM (29.3).|Current smoker (%): No-T2DM (39) > T2DM (23); Alcohol use, past year (%): T2DM (46) < No-T2DM (52).
Melnik, 2006 [24]	Incl. ≥18 y; PR; NYC resident|Excl. NR	64% aged 18–44 y; women 55.8%; >HS 33.8%; U.S.-born 52.9%; nonsmokers 70.8%; T2DM prev. 11.3%
**Mexican American Adults**		
Carrera et al., 2007 [20]	Incl. ≥18 y|Excl. Preg./BF; extreme EI; missing data	Age: men 36.1 vs. women 38.7 y; WC > cutoff: men 34.4% vs. women 70%; smoking: men 26.8% vs. women 13.9%; OW: men 24.5% vs. women 36%; OB: men 47.2% vs. women 38%; edu/income similar
Reininger et al., 2017 [25]	Incl. ≥18 y|Excl. Preg./BF; extreme EI; missing data	≥36 y 68.4%; women ~55%; >8 y edu 72.0%; OW/OB 85.7%; T2DM 24.7%; Spanish pref. 68.0%; 1st-gen 60.3%
**Hispanic Women**		
Arias-Gastélum et al., 2021 [26]	Incl. ≥18 y; Hispanic; BMI ≥ 27; T2DM/prediabetes|Excl. Recent cancer tx; psych hosp.; wt-loss meds; preg./BF	Age 44 y; BMI 36.4 kg/m^2^; OB 86%; WC 115.4 cm; FBG 135 mg/dL; HbA1c 6.5%; sugar/fat and meat/snack DPs associated with younger age
**Multicultural Hispanic U.S. Adults**		
Osborn & Haemer, 2023 [21]	Incl. ≥18 y; Mexican American or other Hispanic|Excl. Missing recalls or sociodemographic	Women 51.7%; income ≥$35 k 57.8%; ≤HS 34.6%; ≥some college 43.6%; U.S.-born 49.1%; ≥10 y U.S. 40.3%; Mexican Americans: 62.3%; other Hispanic origin: 37.7%

Incl., inclusion; Excl., exclusion; y, years; SDA, Seventh-day Adventist; PR, Puerto Rican; DP, dietary pattern; Q5, highest quantile; T3, highest tertile; Veg, vegetarian; non-veg, non-vegetarian; PBD, plant-based diet; T2DM, type 2 diabetes mellitus; No-T2DM, no type 2 diabetes mellitus; BMI, body mass index; WC, waist circumference; OW, overweight; OB, obese; EI, energy intake; PA, physical activity; edu, education; HS, high school; FBG, fasting blood glucose; HbA1c, glycated hemoglobin; tx, treatment; psych hosp., psychiatric hospitalization; preg./BF, pregnant or breastfeeding; NR, not reported; 1st-gen, first-generation immigrant; Spanish pref., Spanish language preference. ^†^ Acculturation score: higher values indicate greater acculturation.

**Table 3:** Dietary patterns and cardiometabolic outcomes in Hispanic/Latinx, *n* = 10.

First Author, Year	Objective	Analysis/Sample/ Outcomes	Dietary Assessment and Pattern Method	Dietary Patterns	Key Results	Conclusions
**Seventh-day Adventist Hispanic/Latinx Adults**						
Singh et al., 2019 [22]	Examine whether plant-based dietary (PBD) patterns are associated with adiposity	Cross-sectional; *n* = 74 SDA Hispanic/Latinx adults (AMEN Study); outcomes: BMI, WC, SBP, DBP	24 h recall plus EPIC-Oxford–adapted FFQ (30-day intake)	2 DPs: (1) Non-vegetarian; (2) PBD (pescatarian, lacto-ovo, strict vegetarian)	PBD vs. non-vegetarian: BMI 24.5 vs. 27.9 kg/m^2^; WC 88.4 vs. 95.2 cm; BP ns	Higher plant-based intake associated with healthier adiposity markers
Alexander et al., 1999 [17]	Compare CVD and T2DM risk factors between SDA vegetarians and Catholic omnivores	Cross-sectional; *n* = 74 SDA vs. *n* = 45 Catholic Hispanic adults; outcomes: BMI, WC, SBP, DBP, TG, glucose, HDL-C, LDL-C	FFQ (3-month intake)	2 DPs: (1) Vegetarians (lacto-ovo/strict); (2) omnivores	Vegetarians vs. omnivores: BMI 27.2 vs. 31.4 kg/m^2^; WC 89.2 vs. 99.3 cm; SBP 109.8 vs. 117.1 mmHg; TG 152 vs. 229 mg/dL; glucose 88.9 vs. 103.4 mg/dL; HDL-C 44.5 vs. 39.9 mg/dL	SDA vegetarians exhibited lower cardiometabolic risk
Jaceldo-Siegl et al., 2019 [23]	Characterize PBD patterns and examine BMI associations	Cross-sectional; *n* = 3475 Hispanic/Latinx SDAs (AHS-2); outcomes: BMI	1-year FFQ validated with six 24 h recalls	2 DPs: (1) non-vegetarian; (2) PBD (vegetarian, pesco-vegetarian, semi-vegetarian)	BMI (% difference vs. non-vegetarian): vegan −14.3%; vegetarian −8.2%; pesco-vegetarian −4.2%; semi-vegetarian −2.96% (ns)	Greater adherence to PBD strongly associated with lower BMI
**Puerto Rican Adults**						
Noel et al., 2009 [18]	Identify dietary patterns and associations with metabolic syndrome (MetS)	Cross-sectional; *n* = 1167 PR adults (BPRHS); outcomes: MetS	12-month FFQ validated with plasma carotenoids; Method: factor analysis	3 DPs: (1) Meat/processed and French fries (“Meats”); (2) Traditional rice/beans (“Traditional PR”); (3) Sweets/sugary beverages and dairy desserts (“Sweets”)	MetS prevalence: Traditional PR > Sweets > Meats (74.5% > 64.9% > 62.1%); Meat Pattern: OR 1.2 [0.76–2.0]; ↑ DBP; Traditional PR: OR 1.7 [1.04, 2.7]; Sweets: OR 1.8 [1.03, 3.3], ↓ HDL-C; ↓ fasting glucose	Traditional PR and sweets patterns linked to higher MetS risk and lower HDL, while the Meat pattern linked to ↑ DBP risk
Del Campo et al., 2020 [19]	Derive dietary pattern scores by diabetes status and examine 2-year CMR change	Cross-sectional plus prospective; *n* = 703 PR adults (BPRHS); outcomes: HDL-C, TG, LDL-C, SBP, WC	1-year FFQ; Method: RRR	2 DPs: ^a^ (1) Diabetes DP Score; (2) No-diabetes DP Score	Cross-sectional: Diabetes DP score ↑ WC; No-diabetes DP score ↓ HDL-C 2-year follow-up: WC ↑ in both groups; Diabetes DP score ↓ LDL-C −4.44 mg/dL [−8.35, −0.54]; No-diabetes DP score ↑ HDL-C 1.03 mg/dL [1.01, 1.05]	Baseline dietary patterns did not predict 2-year CMR change, but among participants, those without diabetes were more likely to be in the highest tertile of the DP score
Melnik, 2006 [24]	Assess fat-related dietary behaviors by diabetes status among PR adults	Cross-sectional; *n* = 1304 PR adults (NYC BRFSS); outcomes: T2DM	BRFSS fat-related diet habits questionnaire (3-month intake)	Composite fat-related diet score (1–4-point scale, with higher scores = ↑ fat intake). Subscales: (1) Avoid fat as flavoring: (2) Avoid fried foods: (3) Modify meats to be low fat: (4) Substitute fat-modify products	Diabetes associated with lower fat intake: β −0.27, *p* = 0.01; interactions: diabetes × family history β 0.19, *p* = 0.03; diabetes × weight β 0.26, *p* = 0.02; diabetes × exercise β −0.23, *p* = 0.01	Adults with diabetes practiced more meat-modification and fat-lowering behaviors. Obesity and family history predicted higher fat intake
**Mexican American Adults**						
Carrera et al., 2007 [20]	Examine dietary patterns and central obesity among Mexican Americans	Cross-sectional; *n* = 659 Mexican American adults (NHANES 2001–2002); outcomes: BMI, WC	24 h recall; Method: cluster analysis	4 DPs: (1) Poultry/alcohol; (2) Milk/baked goods; (3) Traditional Mexican; 4) Meat	BMI similar across patterns at ~28 kg/m^2^; WC (cm): women: meat 95.2 > poultry 94.2 > traditional Mexican 93.1 > milk/baked 92.0; men: traditional Mexican 97.8 > poultry 95.8 > milk/baked 95.6 > meat 94.2	All four DPs had high mean BMI. Women in all DPs showed a mean WC above the cutoff of 88 cm.
Reininger et al., 2017 [25]	Examine healthy and unhealthy dietary patterns and diabetes prevalence in Mexican Americans	Cross-sectional; *n* = 1250 Mexican American adults (Cameron County Hispanic Cohort); outcomes: BMI, T2DM	SPAN-adapted 24 h recall plus Method: HEI and UEI	2 DPs: ^b^ (1) Healthy Eating Index, (2) Unhealthy Eating Index	HEI ↑ with age, Spanish preference, first generation; UEI ↑ with acculturation and second generation; no association with BMI or diabetes	Less acculturation (Spanish-preferring, 1st-gen, older adults) → ↑ HEI + ↓ UEI; More acculturation (English-preferring, women, 2nd-gen) → ↑ UEI + ↓ HEI; DPs not associated with BMI or diabetes prevalence
**Hispanic Women**						
Arias-Gastélum et al., 2021 [26]	Identify dietary patterns and associations with glycemic risk	Cross-sectional; *n* = 191 Hispanic women with diabetes or prediabetes; outcomes: FBG, HbA1c	3-mo intake (SWFFQ); Method: factor analysis	6 DPs: (1) Sugar/Fat-laden (“Western”); (2) Plant/Fish (“Mediterranean-like”); (3) Soups/Starch (“Traditional.”); (4) Meat/Snacks (“High-fat, processed meat pattern”); (5) Beans/Grains (“Mixed “healthy + processed” pattern”); (6) Eggs/Dairy	Sugar and fat-laden and meat and snacks patterns associated with younger age: β −0.23 and −0.30; plant and fish pattern associated with higher FBG: β 0.15; no associations with BMI, WC, or HbA1c	Sugar/fat-laden and meat/snack DPs were negatively associated with age. The plant/fish DP, unexpectedly, was significantly associated with ↑ FBG
**Multicultural Hispanic U.S. Adults**						
Osborn and Haemer, 2023 [21]	Identify dietary patterns among U.S. Hispanic adults and cardiometabolic associations	Cross-sectional; *n* = 2049 Hispanic adults (NHANES 2013–2018); outcomes: BMI, T2DM, body fat, MI, CHD, HDL-C, LDL-C, TG, SBP, DBP	Two 24 h recalls; Methods: factor analysis	3 DPs: ^c^ (1) Solid fats and refined carbohydrates, (2) Vegetables, (3) Plant-based	Solid fats and refined carbohydrates: ↑ BMI β 1.07 [0.14, 1.99], ↓ HDL-C β −4.53 [−7.03, −2.03], ↓ SBP β −2.47 [−4.89, −0.59]; Vegetables: ↓ body fat percentage β −1.57 [−2.74, −0.39], ↓ HDL-C at moderate intake β −2.62 [−4.79, −0.47]; Plant-based: no associations	Energy-dense patterns linked to worse cardiometabolic health. Solid fats/refined carbs DP was associated with ↑ BMI, ↓HDL-C; the vegetable DP was linked to ↓ body fat and ↓ HDL-C at moderate intake, and the DP 3 showed no significant associations with CMR factors

^a^ Diabetes DP score (higher intakes of pizza, white bread, processed meats, sweetened beverages, and soft drinks; lower intakes of nuts and seeds, reduced-fat dairy products, and starchy vegetables) and No-diabetes DP score (higher intakes of white bread, solid fats, sweet baked goods, processed meats, and rice; lower intakes of nuts and seeds, poultry, and water). ^b^ Healthy Eating Index (HEI): higher intakes of beans, eggs, fruit, fruit juice, vegetables, salads, whole grains, and chicken or fish; and Unhealthy Eating Index (UEI): higher intakes of baked goods, French fries and chips, fried meats, frozen desserts, processed meats, regular soda, sweetened beverages, and white bread. AHS-2, Adventist Health Study–2; AMEN, Adventist Multiethnic Nutrition Study; BMI, body mass index (kg/m^2^); BP, blood pressure; BPRHS, Boston Puerto Rican Health Study; BRFSS, Behavioral Risk Factor Surveillance System; CHD, coronary heart disease; CMR, cardiometabolic risk; DBP, diastolic blood pressure (mmHg); DP, dietary pattern; EPIC, European Prospective Investigation into Cancer and Nutrition; FBG, fasting blood glucose (mg/dL); FFQ, food frequency questionnaire; HbA1c, hemoglobin A1c (%); HDL-C, high-density lipoprotein cholesterol (mg/dL); HEI, Healthy Eating Index; LDL-C, low-density lipoprotein cholesterol (mg/dL); MetS, metabolic syndrome; MI, myocardial infarction; NHANES, National Health and Nutrition Examination Survey; ns, not significant; OR, odds ratio; PBD, plant-based diet; PR, Puerto Rican; RRR, reduced rank regression; SBP, systolic blood pressure (mmHg); SDA, Seventh-day Adventist; SPAN, School Physical Activity and Nutrition dietary recall instrument; SWFFQ, Southwestern Food Frequency Questionnaire; TG, triglycerides (mg/dL); T2DM, type 2 diabetes mellitus; UEI, Unhealthy Eating Index; WC, waist circumference (cm); β, beta coefficient. ^c^ Solid fats/refined carbohydrates: solid fats, refined grains, cheese, added sugars, and tomato products; Vegetables: red and orange vegetables, green vegetables, and other vegetables; Plant-based diet (PBD): soy foods, nuts and seeds, whole fruits, and red and orange vegetables.

## Data Availability

The data extracted during the current study are available from the corresponding author.

## Supplementary Materials

The following supporting information can be downloaded at: https://www.mdpi.com/article/10.3390/nu18020220/s1. Table S1. Search MeSh Terms; Supplemental S2. Newcastle-Ottawa Scale adapted for cross-sectional studies; Supplemental S3. Supplemental List of Studies Assessed for Eligibility.

## Abbreviations

The following abbreviations are used in this manuscript:

AHS-2	Adventist Health Study–2
AMEN	Adventist Multiethnic Nutrition Study
BMI	body mass index (kg/m^2^)
BP	blood pressure (mmHg)
BPRHS	Boston Puerto Rican Health Study
BRFSS	Behavioral Risk Factor Surveillance System
CHD	coronary heart disease
CI	confidence interval
CMR	cardiometabolic risk
CMD	cardiometabolic diseases
CVD	cardiovascular disease
DBP	diastolic blood pressure (mmHg)
DP	dietary pattern
DASH	Dietary Approaches to Stop Hypertension
EPIC	European Prospective Investigation into Cancer and Nutrition
FBG	fasting blood glucose (mg/dL)
FFQ	food frequency questionnaire
HbA1c	hemoglobin A1c (%)
HCHS/SOL	Hispanic Community Health Study/Study of Latinos
HDL-C	high-density lipoprotein cholesterol (mg/dL)
HEI	Healthy Eating Index
LDL-C	low-density lipoprotein cholesterol (mg/dL)
MetS	metabolic syndrome
MI	myocardial infarction
NHANES	National Health and Nutrition Examination Survey
NOS	Newcastle–Ottawa Scale
ns	not significant
NYC	New York City
OR	odds ratio
OW/OB	overweight/obese
PBD	plant-based diet
PICO	Population, Intervention/Exposure, Comparison, Outcome
PR	Puerto Rican
RCT	randomized controlled trial
RoB	risk of bias
RRR	reduced rank regression
SDA	Seventh-day Adventist
SBP	systolic blood pressure (mmHg)
SES	socioeconomic status
SPAN	School Physical Activity and Nutrition
SWFFQ	Southwestern Food Frequency Questionnaire
T2DM	type 2 diabetes mellitus
TG	triglycerides (mg/dL)
U.S.	United States
UEI	Unhealthy Eating Index
WC	waist circumference (cm)
WHO	World Health Organization
