# Supplementary material for: Plant-Forward Dietary Approaches to Reduce the Risk of Cardiometabolic Disease Among Hispanic/Latinx Adults Living in the United States: A Narrative Review
Source: Nutrients. 2026 Jan 10;18(2):220. doi: 10.3390/nu18020220 (PMC12845153; doi:10.3390/nu18020220)
Supplement: Supplementary file 1 [file nutrients-18-00220-s001.zip › nutrients-4087856-supplementary.pdf]

Supplemental Materials

1. **Table S1.** Search MeSh Terms
2. **Supplemental S2.** Newcastle-Ottawa Scale adapted for cross-sectional studies
3. **Supplemental S3.** Supplemental List of Studies Assessed for Eligibility

Table 1. Search MeSh Terms

PICO Concept	Keyword	MeSh Terms on Medline
P- Hispanic/Latinx adults aged 18 years or older living in the U.S..	Hispanic, Latinos, adults	("hispanic or latino"[MeSH Terms] OR ("hispanic"[All Fields] AND "or"[All Fields] AND "latino"[All Fields]) OR "hispanic or latino"[All Fields] OR "hispanic"[All Fields] OR "hispanics"[All Fields] OR ("hispanic or latino"[MeSH Terms] OR ("hispanic"[All Fields] AND "or"[All Fields] AND "latino"[All Fields]) OR "hispanic or latino"[All Fields] OR "latino"[All Fields] OR "latinos"[All Fields]) OR ("hispanic or latino"[MeSH Terms] OR ("hispanic"[All Fields] AND "or"[All Fields] AND "latino"[All Fields]) OR "hispanic or latino"[All Fields] OR "latinx"[All Fields] OR "latinxs"[All Fields])) AND ("adult"[MeSH Terms] OR "adult"[All Fields] OR "adults"[All Fields] OR "adult s"[All Fields])
I-Defined dietary patterns identified using a priori, a posteriori, or hybrid methods	Dietary pattern, diet pattern, dietary index	((("dietary"[All Fields] AND "patterns"[All Fields]) OR "dietary patterns"[All Fields] OR ("diet"[MeSH Terms] OR "diet"[All Fields]))OR "vegetarian diet"[All Fields] OR "vegetarians"[MeSH Terms] OR "vegetarians"[All Fields] OR "vegetarian"[All Fields] OR "diet, vegan"[MeSH Terms] OR ("diet"[All Fields] AND "vegan"[All Fields]) OR "vegan diet"[All Fields] OR "veganism"[All Fields] OR "vegans"[MeSH Terms] OR "vegans"[All Fields] OR "vegan"[All Fields]
C - Differences between dietary pattern groups	n/a	n/a
O- Associations with at least one cardiometabolic risk (CMR) factor.	Cardiometabolic risk factors, Cardiometabolic syndrome, cardiometabolic	("cardiometabolic"[All Fields] OR "cardiometabolically"[All Fields]) AND ("disease"[MeSH Terms] OR "disease"[All Fields] OR "diseases"[All Fields] OR "disease s"[All Fields] OR "diseased"[All Fields])))

Supplemental S2. Newcastle-Ottawa Scale adapted for cross-sectional studies

Selection: (Maximum 5 stars)

1) Representativeness of the sample:
- a) Truly representative of the average in the target population. * (all subjects or random sampling)
- b) Somewhat representative of the average in the target population. * (non-random sampling)
- c) Selected group of users.
- d) No description of the sampling strategy.

2) Sample size:
- a) Justified and satisfactory. *
- b) Not justified.

3) Non-respondents:
- a) Comparability between respondents and non-respondents' characteristics is established, and the response rate is satisfactory. *
- b) The response rate is unsatisfactory, or the comparability between respondents and non-respondents is unsatisfactory.
- c) No description of the response rate or the characteristics of the responders and the non-responders.

4) Ascertainment of the exposure (risk factor):
- a) Validated measurement tool. **
- b) Non-validated measurement tool, but the tool is available or described. *
- c) No description of the measurement tool.

Comparability: (Maximum 2 stars)

1) The subjects in different outcome groups are comparable, based on the study design or analysis. Confounding factors are controlled.
- a) The study controls for the most important factor (select one). *
- b) The study control for any additional factor. *

Outcome: (Maximum 3 stars)

1) Assessment of the outcome:
- a) Independent blind assessment. **
- b) Record linkage. **
- c) Self report. *
- d) No description.

2) Statistical test:

a) The statistical test used to analyze the data is clearly described and appropriate, and the measurement of the association is presented, including confidence intervals and the probability level (p value). *

b) The statistical test is not appropriate, not described or incomplete.

Scores for cross-sectional studies:

- Very good quality: 9-10 points
- Good quality: 7-8 points
- Satisfactory quality: 5-6 points
- Unsatisfactory quality: 0 to 4 points

This scale was adapted from the Newcastle-Ottawa Quality Assessment Scale for cohort studies created by Abesig J, et al., (2020) [1]

References

1. Abesig, J., et al., Prevalence of viral hepatitis B in Ghana between 2015 and 2019: A systematic review and meta-analysis. PloS one, **2020**. **15**(6): p. e0234348.

Supplemental S3. List of Studies Assessed for Full Eligibility

1. Diet quality and its association with cardiometabolic risk factors vary by Hispanic and Latino ethnic background in the Hispanic Community Health Study/Study of Latinos
2. Association of Dietary Patterns with Cardiovascular Disease Risk Factors in Mexican Adults: Insights from a Cross-Sectional Descriptive Study
3. **A study of dietary patterns in the Mexican-American population and their association with obesity**
4. A Posteriori dietary patterns, insulin resistance, and diabetes risk by Hispanic/Latino heritage in the HCHS/SOL cohort
5. Body mass of US Hispanics/Latinos from the Hispanic community health study/study of Latinos (HCHS/SOL): How do diet quality and sedentary time relate? If obesity yes, but if body composition no.
6. Psychosocial stress is associated with obesity and diet quality in Hispanic/Latino adults only if talk about obesity and diet quality.
7. Association Between Ultra-Processed Food Intake and Metabolic Profile and Risk of Type 2 Diabetes and Cardiovascular Disease in US-Residing Cohorts of Hispanic/Latino and White Adults if stratified.
8. **Dietary Patterns Associated with Cardiometabolic Risk for Puerto Ricans with and Without Diabetes, and the Psychosocial Health Correlates of Adherence to the Disease-Related Patterns.**
9. DASH diet and prevalent metabolic syndrome in the Hispanic Community Health Study/Study of Latinos.
10. Dietary factors, gut microbiota, and serum trimethylamine-N-oxide associated with cardiovascular disease in the Hispanic Community Health Study/Study of Latinos.
11. **Dietary patterns with healthy and unhealthy traits among overweight/obese Hispanic women with or at high risk for type 2 diabetes.**
12. Food-group and nutrient-density intakes by Hispanic and Latino backgrounds in the Hispanic Community Health Study/Study of Latinos background.
13. Cultural and dietary factors influencing traditional Latino meal patterns: Findings from focus group discussion background.
14. Association of dysfunctional eating patterns and metabolic risk factors for cardiovascular disease among Latinos.
15. **Dietary patterns and their association with cardiometabolic biomarkers and outcomes among Hispanic adults: a cross-sectional study from the National Health and Nutrition Examination Survey (2013–2018).**
16. Dietary patterns and years living in the United States by Hispanic/Latino heritage in the Hispanic Community Health Study/Study of Latinos (HCHS/SOL) background.
17. Higher-protein intake and physical activity are associated with healthier body composition and cardiometabolic health in Hispanic adults.
18. **A traditional rice and beans pattern is associated with metabolic syndrome in Puerto Rican older adults**
19. Acculturation and sociocultural influences on dietary intake and health status among Puerto Rican adults in Massachusetts.
20. Community resource utilization, psychosocial health, and sociodemographic factors associated with diet and physical activity among low-income obese Latino immigrants.
21. **Plant-Based Diets Are Associated with Lower Adiposity Levels Among Hispanic/Latino Adults in the Adventist Multi-Ethnic Nutrition (AMEN) Study.**
22. Healthy dietary patterns and risk of cardiovascular disease in US Hispanics/Latinos: the Hispanic Community Health Study/Study of Latinos (HCHS/SOL).
23. Association of the DASH dietary pattern with insulin resistance and diabetes in US Hispanic/Latino adults: results from the Hispanic Community Health Study/Study of Latinos (HCHS/SOL).

24. Healthy dietary patterns are associated with the gut microbiome in the Hispanic Community Health Study/Study of Latinos.
25. **Plant-based diets in hispanic/latino adult adventists in the United States and their association with body mass index.**
26. Healthful eating patterns, serum metabolite profile and risk of diabetes in a population-based prospective study of US Hispanics/Latinos.
27. Comparing Acculturation Models in Evaluating Dietary Habits Among Low-Income Hispanic Women.
28. Dietary patterns in blacks and Hispanics with diagnosed diabetes in New York City's South Bronx only if stratified.
29. How Well Do U.S. Hispanics Adhere to the Dietary Guidelines for Americans? Results from the Hispanic Community Health Study/Study of Latinos.
30. Diet Quality and Eating Practices among Hispanic/Latino Men and Women: NHANES 2011–2016.
31. Intake and Food Sources of Macronutrients Among Older Hispanic Adults: Association with Ethnicity Acculturation, and Length of Residence in The United States.
32. Dietary Intake Patterns and Acculturation Levels of Hispanic Immigrant Men: A Pilot Study
33. Neighbourhood immigrant acculturation and diet among Hispanic female residents of New York City.
34. Food-Insecure Dietary Patterns Are Associated with Poor Longitudinal Glycemic Control in Diabetes: Results from the Boston Puerto Rican Health Study.
35. **Fat-related dietary behaviors of adult Puerto Ricans, with and without diabetes, in New York City.**
36. Hispanic Women in EFNEP Have Low Adherence with Dietary Guidelines Regardless of Acculturation Level.
37. **Risk factors for cardiovascular disease and diabetes in two groups of Hispanic Americans with differing dietary habits.**
38. Feasibility and acceptability of a Mediterranean-style diet intervention to reduce cardiovascular risk for low-income Hispanic American women.
39. Feasibility and Acceptability of a Clinic-based Mediterranean-style Diet Intervention to Reduce Cardiovascular Risk for Hispanic Americans with Type 2 Diabetes.
40. En Balance Participants Decrease Dietary Fat and Cholesterol Intake as Part of a Culturally Sensitive Hispanic Diabetes Education Program.
41. The Gut Microbiome Modifies the Association Between a Mediterranean Diet and Diabetes in USA Hispanic/ Latino Population.
42. Dietary Patterns in Mexican Adults Are Associated with Risk of Being Overweight or Obese.
43. Similarities and Dissimilarities in Diet Quality Differences by Acculturation Level between Mexican Americans and Other Hispanic Americans: National Health and Nutrition Examination Survey 2015–2018.
44. Examining the Diet of Post-Migrant Hispanic Males Using the Precede-Proceed Model: Predisposing, Reinforcing, and Enabling Dietary Factors.
45. A Meat, Processed Meat, and French Fries Dietary Pattern Is Associated with High Allostatic Load in Puerto Rican Older Adults.
46. **Healthy eating patterns associated with acculturation, sex and BMI among Mexican Americans.**
47. Diets high in subsidized foods and chronic kidney disease in Hispanic communities in the United States: the Hispanic Community Health Study/Study of Latinos.
48. Examining Generalizability of Nutrient-Based Food Patterns and Their Cross-Sectional Associations with Cardiometabolic Health for Hispanic/Latino Adults in the US: Results from the National Health and Nutrition Examination Survey (NHANES) and the Hispanic Community Health Study/Study of Latinos (HCHS/SOL).

49. Dietary Acculturation Is Associated with Altered Gut Microbiome, Circulating Metabolites, and Cardiovascular Disease Risk in US Hispanics and Latinos: Results From HCHS/SOL.
50. Dietary inflammatory index and cardiovascular disease risk in Hispanic women from the Women's Health Initiative.
51. Lifestyle factors and genetic variants associated to health disparities in the Hispanic population.
52. Association of Diet Quality Indices with Longitudinal Changes in Kidney Function in U.S. Hispanics/Latinos: Findings from the Hispanic Community Health Study/Study of Latinos (HCHS/SOL).
53. Associations of sodium and potassium intake with chronic kidney disease in a prospective cohort study: findings from the Hispanic Community Health Study/Study of Latinos, 2008–2017.
54. Diversifying nutritional sciences—dietary practices and gut bacteria in individuals of Latino and Hispanic ancestry.
